# Penile Rehabilitation and Neuromodulation

**DOI:** 10.1100/tsw.2009.86

**Published:** 2009-07-27

**Authors:** Fernando Facio, Arthur L. Burnett

**Affiliations:** ^1^ Department of Urology, The Johns Hopkins Hospital, Baltimore, MD, USA; ^2^ Department of Urology, The James Buchanan Brady Urological Institute, The Johns Hopkins Medical Institutions, Baltimore, MD, USA

**Keywords:** prostate cancer, radical prostatectomy, erectile dysfunction, penile erectile

## Abstract

Erectile dysfunction (ED) following treatment for clinically localized prostate cancer, particularly radical prostatectomy (RP), is a major quality of life issue that remains unsatisfactorily addressed. With the introduction and use of cavernous nerve–sparing procedures over the past 25 years, many men recover erections postoperatively that enable sexual intercourse unlike in the prior surgical era, when permanent ED postoperatively was certain. Despite this advance, 26–100% of these patients may never recover normal erectile function (EF). Recent advances in the understanding of ED after RP have stimulated great attention to develop penile rehabilitation programs and neuromodulation. The purpose of penile rehabilitation is to prevent adverse corpus cavernosal tissue structural alterations and thereby maximize the chances of recovering functional erections. Rehabilitation programs are common in clinical practice, but there is no definitive evidence to support their efficacy. Neuromodulation represents another strategy for promoting erection recovery postoperatively. This therapy involves the application of neuroprotective interventions, conceivably targeting biological elements involved in the erection response that are affected by neuropathic injury. Well-conducted, controlled trials with adequate follow-up are required in order to determine the erection preservative benefits of these therapeutic strategies. The purpose of this essay is to describe the mechanisms related to post-RP ED, assess the need for penile rehabilitation and neuromodulation following surgery, and analyze the basic science and clinical trial evidence associated with these applications for preserving EF following prostate cancer treatment.

### INTRODUCTION

Prostate cancer has emerged from an often trivialized medical condition, relegated to older men and thought to exert little lifetime consequence, to a disease state of major importance. The significance of the disease has increased in large part because of its dramatic stage migration in the modern prostate-specific antigen (PSA) era, and this is typified by increasingly early clinical stage diagnoses and diagnoses made increasingly in young men[1]. Radical prostatectomy (RP) is a major option for the treatment of clinically localized prostate cancers with excellent long-term results. RP reduces disease-specific mortality, overall mortality, and the risks of metastasis and local progression. In a randomized, controlled trial comparing

RP to watchful waiting, the absolute reduction in the risk of death after 10 years was small, but the reductions in the risks of metastasis and local tumor progression were substantial[2]. Patients are therefore increasingly likely to choose active treatment, provided that morbidity such as erectile dysfunction (ED) is acceptable.

Post-RP ED is a common problem, with reported rates of 26–100% for complete ED and 16–48% for partial ED[3]. The surgeon's experience and volume of surgeries performed are conceivably dominant factors influencing these outcomes[4]. Walsh advanced the understanding of pelvic neuroanatomy and pioneered the development of a surgical technique by which the entire prostate could be removed, while preserving the anatomical integrity of the autonomic nerves surrounding the gland. The procedure is termed nerve-sparing radical prostatectomy (NSRP). Despite current surgical improvements in techniques used in RP, which include anatomical preservation of the penile nerves, many men experience a delay or failure in achieving full recovery of natural penile erection postoperatively[1].

Recently, robotic RP has been introduced with enthusiasm[5]. The proponents of this technique tout conceivable advantages of this procedure, including minimal invasiveness, shorter length of hospitalization, and faster postoperative recovery[6,7,8]. They emphasize the three-dimensional visualization and magnification power as an advantage of the robotic device[9]. It is postulated that under magnification, the additional nerve fibers running on the lateral surface of the prostate are better identified and preserved, and consequently, this leads to improved postoperative potency[5]. Although results of patients who have undergone robotic RP appear to be at least equal to laparoscopic RP or open RP, assessment of long-term oncologic and functional outcomes will be required in order to establish the role and efficacy of robotic RP.

It is recognized that other interventions for prostate cancer, including cryosurgery, external beam radiotherapy (EBRT), brachytherapy, and hormonal ablation, all have the potential to affect penile erections[1]. Sanda et al.[10] demonstrated that 53 and 57% of the patients who had undergone EBRT and brachytherapy, respectively, complained about some degree of ED after 24 months. As after RP, the full erectile function (EF) recovery rate is variable, ranging between 24% after treatment with cryosurgery[11,12], 36–63% after EBRT[13,14], and 18% after brachytherapy[3,15]. Further worsening in the sexual function score was observed in those groups in which hormonal therapy was administered[16]. The variability of findings suggests that there probably are inconsistencies in ED definition, assessment, and reporting in this literature.

New directions to manage ED in association with prostate cancer treatment would seemingly aim to surpass the limitations of conventional management options. Current options, which include on-demand oral medications, local penile treatments, and penile prosthesis surgery, are limited by requiring repetitive administrations or removing the opportunity for normal erections. Penile rehabilitation and neuromodulation are two such new directions that are strategically intended to facilitate the preservation of natural EF for this clinical condition. In keeping with the notion that ideal therapy achieves natural and normal erections, the ultimate goal in managing ED is to recover this exact level of functional ability postoperatively. From this perspective, this essay is intended to review current proposals about penile rehabilitation and neuromodulation, specifying the post-RP ED paradigm, and to present several major biomedical research directions that may foster clinically applicable therapeutics for ED in this clinical context for the future.

### PATHOPHYSIOLOGY

ED is clinically evident immediately after RP. This result is associated with cavernous nerve (CN) injury during surgery. Even for bilateral CN preservation, some trauma is delivered to the nerves. Common causes include mechanical traction, thermal damage due to electrocautery use, nerve ischemia due to vascular injury, and local inflammatory effects associated with surgical trauma[17]. The result is temporary inactivity known as neuropraxia. It is more than likely that functional or structural damage of the penile nerve supply results in subsequent deterioration of cavernosal tissue physiology and, consequently, induction of penile neuropathy[18]. This insult results in reduced or lack of nitric oxide (NO) release from erection-producing CN and, ultimately, a continuous state of constriction of the penile vascular smooth muscle. The hypoxic state that follows may lead to the development of fibrosis within the corpora cavernosa[19], leading to cavernous veno-occlusive dysfunction, the major etiology of post-RP ED[17]. Decreased formation of NO and cyclic guanosine monophosphate (cGMP) in cavernosal tissue is believed to play a role in the apoptotic and fibrotic mechanisms that limit EF recovery[20].

Mechanisms of vascular injury have also been proposed as a possible basis for post-RP ED[21]. Vascular abnormalities take two forms: arterial insufficiency and venous leakage[22]. The hypothesis to explain the arterial component is that injury is sustained by accessory pudendal arteries (APA) during the surgery. The incidence of damage of the lateral and apical APA during the surgery ranges from 4–75%[23]. Rogers et al.[24] observed a trend toward a statistically significant difference in post-RP ED recovery in the group who had APA preservation (93%) compared with those who did not (70%). The second form refers to venous leakage or corporal veno-occlusive dysfunction (CVOD), and is the consequence of neuropraxia/neurotomy[20]. Subsequent to the neural damage, apoptosis and fibrosis occur in the penis, which pathologically result in CVOD. This condition occurs because of the inability of the cavernosal smooth muscle cell mass to compress the subtunical veins sufficiently and prevent leakage of blood out of the corpora cavernosa during tumescence[21].

### ERECTILE DYSFUNCTION

ED management has moved forward substantially in the past 25 years with the introduction of a host of remarkable therapeutic options. Not too long ago, management for ED was directed largely towards psychosocial or hormonal factors, in accordance with the presumption that these were causative conditions for the disorder. Hence, therapy was generally administered in the forms of psychoanalysis, sex therapy, and hormonal interventions. If such management did not work, alternative strategies were used, ranging from herbal supplements presumed to enhance sexual performance to mechanical devices[25].

Since the introduction of sildenafil citrate (Viagra®) in the late 1990s, phosphodiesterase type 5 inhibitors (PDE5i) have become first-line therapy for ED[26]. The advent of PDE5i revolutionized ED treatment with an average successful sexual intercourse rate of 60–70% in the general ED population[27]. Vacuum erection devices (VED) and intracavernosal injections (ICI) commonly represent second-line therapies. Penile prosthesis surgery is designated as third-line therapy for ED treatment. Principles of this process include application of options initially that are least invasive, easily administered, and generally less expensive, while subsequently escalating management as needed. On the other hand, patient motivation and treatment compliance must be considered before instituting any therapy[28,29].

### PENILE REHABILITATION

Penile rehabilitation is defined as the use of any drug or device at the time of or after any intervention for prostate cancer that serves to maximize EF recovery or preservation subsequently[30]. The purpose of penile rehabilitation is to prevent corpus cavernosal tissue structural alterations caused by the prostate cancer treatment and preserve normal erection biologic mechanisms[30]. The clinical strategy of postoperative penile rehabilitation after RP arose from the concept that induced early sexual stimulation and augmented blood flow to the penis would facilitate the return of natural EF and resumption of medically unassisted sexual activity. The crucial questions, therefore, are how to rehabilitate and when to rehabilitate. Emerging data from animal studies suggest that rehabilitation is possible[31,32].

Several clinical trials support the use of ICI, PDE5i, intraurethral alprostadil, and VED in penile rehabilitation programs. Although there is currently no consensus regarding the implementation of penile rehabilitation programs, its initiation time, the frequency of application, the type of vasoactive agents, and the dose regimen to be used, a number of recent studies have reported various approaches. PDE5i and ICI are more commonly used in rehabilitation programs. Regular use is proffered, starting as early as possible (from the day of catheter removal or during the first month after surgery), although there are no approved guidelines[32,33]. Recently, researchers have reported a time interval of 9 months to regain potency with nightly dosing of sildenafil therapy[34] (Table 1).

**TABLE 1**
**Conventional Therapies Applied for Penile Rehabilitation**

| | **Putative Mechanisms of Action** |
|---|---|
| PDE5i (oral) | Antiapoptosis |
| | Edothelial cell protection |
| PGE1 (intracavernosal or intraurethral) | Cavernosal oxygenation |
| | Endothelial cell protection |
| | Reduced collagen deposition |
| VED | Endothelial cell protection |
| | Antifibrosis |

### Oral Therapy

Although PDE5i are popular and offer an average successful sexual intercourse rate of 60–70% in general ED groups[27], the rationale and mechanism for their use in penile rehabilitation programs after RP have not been fully elucidated.

In a recent prospective study, Mulhall et al.[35] evaluated the use of an erectogenic pharmacotherapy regimen following RP to determine if it would improve recovery of spontaneous EF. Men with functional preoperative erections who underwent RP were challenged early after surgery with oral sildenafil (rehabilitation group, n = 58). Nonresponders to oral sildenafil were switched to ICI therapy. EF of these patients was compared with men who did not have any rehabilitation (no rehabilitation group, n = 74) at 18 months following RP. Pharmacologic penile rehabilitation in this study resulted in improvements in a number of outcomes, including the proportion of men who had recovery of spontaneous EF, the ability to respond to sildenafil, the time course to respond to sildenafil, the ability to respond to ICI, the percentage of men who had normalization of EF domain scores, the number of men left with less severe ED after RP, and a reduced dose of ICI medication required to obtain an erection rigid enough for penetration[35]. Unfortunately, limitations of this study were a small number of patients included, nonrandomization, and lack of a placebo control group.

Bannowsky et al.[36] evaluated the effect of low-dose sildenafil for rehabilitating EF after NSRP. Forty-three sexually active patients had a NSRP; at 7–14 days after surgery, they had a Rigiscan (Dacomed Corporation, Minneapolis, MN) measurement of nocturnal penile tumescence and rigidity (NPTR). To support the recovery of spontaneous EF, 23 patients with preserved nocturnal erections received sildenafil 25 mg/day at night. A control group of 18 patients were then followed, but had no PDE5i. Among these 43 patients, 41 (95%) had one to five erections during the first night after catheter removal. In the group using daily sildenafil, the mean IIEF-EF score decreased from 20.8 before NSRP to 3.6, 3.8, 5.9, 9.6, and 14.1 at 6, 12, 24, 36, and 52 weeks after NSRP, respectively. In the control group, the respective scores were 21.2, decreasing to 2.4, 3.8, 5.3, 6.4, and 9.3, respectively. There was a significant difference in IIEF-EF score and time to recovery of EF between the groups ($p < 0.001$), with potency rates of 86 vs. 66%. The measurement of NPTR after NSRP showed EF even the “first” night after catheter removal. As suggested by this study, daily low-dose sildenafil leads to a significant improvement in the recovery of EF. Unfortunately, this study does not address the prevalence of nocturnal erections for all the patients in the treatment arm compared with those in the placebo control arm. The study also suffers from selection bias and lack of randomization in receiving treatment.

Raina et al.[19] reported that 50% of patients recovered spontaneous natural erections at 6 months after using the combination of daily sildenafil (50 mg) and either early ICI of prostaglandins E1 (PGE1) (mean 4 µg /2–3 times/week) starting 2 weeks after RP, or 30 U of low-dose trimix (papaverine, phentolamine, PGE1, 2–3 times/week) to promote daily vasodilation. In this study, it was noted that there were no patients who dropped out. This possibly could be due to the allowance of dose titration. Unfortunately, there was no monotherapy group as a control in this study.

Padma-Nathan et al.[37] documented a significant benefit of either 50 or 100 mg of sildenafil administration each night for 9 months, compared with placebo, in men after undergoing bilateral NSRP. In this study, the patients receiving sildenafil for 36 weeks documented a 27% return of spontaneous normal erectile activity compared with 4% in the placebo group. These results suggested a rationale for early prophylaxis with PDE5i in order to promote earlier recovery of EF after NSRP. However, this study has been criticized because the return of spontaneous erections in the placebo group is only 4%, which was very low compared to the other reported series in the literature[19]. It is possible that this study applied a more stringent definition of erection recovery than what has been applied in historical reports. Montorsi et al.[38] demonstrated that nightly use of sildenafil significantly increased the overall quality and quantity of nocturnal erections as recorded by RigiScan (Timm Medical Technologies Inc., Eden Prairie, MN) in men with ED when compared with placebo[38]. The purported mechanisms to explain these results were reduction in postoperative corporal hypoxia, enhanced endothelial function, and possible neurotropic mechanisms. Despite the suggestion of benefit with this regimen, it remains unclear whether the achievement of nocturnal erections equates with successful recovery of sexually stimulated erections.

On the other hand, Montorsi et al.[39] reported recently, in a randomized, double-blind, multicenter, placebo-controlled trial in patients after NSRP using vardenafil, that there was no difference in EF recovery between on-demand PDE5i and nightly PDE5i as rehabilitative treatment. Mean SEP3 (ability to maintain erection for sexual intercourse) rates after the open-label phase were placebo (57.1%), vardenafil nightly (59.8%), and vardenafil on demand (62.6%), respectively. Vardenafil's efficacy when used on demand supports a paradigm shift towards on-demand dosing with PDE5i in this patient group[39]. This is the only properly designed clinical trial comparing daily vs. on-demand PDE5i in post-RP ED published so far. These results remain to be confirmed for other PDE5i and different dosing schedules. However, the likely conclusion may prompt reconsideration of the current clinical practice prescribing daily PDE5i after RP.

The major criticisms of these studies, in general, include lack of clear therapeutic benefits, relatively small number of patients included, and nonrandomized, nonplacebo-controlled design in some of the studies. It remains difficult to recommend any particular penile rehabilitation therapy using PDE5i in combination with others therapies at the present moment. Larger, properly designed, trials are necessary to assess efficacy and safety of this therapy.

### Intraurethral Prostaglandin

In patients who do not respond to or who are unable to use PDE5i because of contraindications, second-line treatment options may be pursued. Intraurethral alprostadil, a prostaglandin E1 (PGE-1) derivative, referred to as MUSE (VIVUS, Inc., Mountain View, CA), is reportedly effective for treating ED[40]. In patients having ED after RP, Raina et al.[41] studied 91 men who had undergone NSRP with a mean follow-up of 6 months. Fifty-six men were treated with MUSE at 125 or 250 µg three times per week for 6 months. MUSE was started 3 weeks after surgery. Thirty-five men representing the control group did not receive any early treatment. Thirty-two percent (18/56) discontinued treatment. Overall, 40% (15/38) at 6 months achieved natural erections sufficient for satisfactory sexual intercourse. All MUSE-treated patients had penile pain. Although there is an indication that there may be value to transurethral PGE1

administration as a rehabilitation strategy, given the small number of studies, the small population sizes studied, and study methodologic concerns, a limited number of conclusions can be made. There is a distinct need to conduct a large, multicenter, randomized, controlled trial to define its role.

### Intracavernosal Injection

ICI of vasoactive agents, conventionally consisting of PGE-1 (alprostadil [Prostin VR, Caverjet, Edex]), papaverine, and phentolamine, has been used very successfully for the treatment of a broad range of causes of ED. Efficacy for erections useful for sexual intercourse rates commonly range between 70 and 90%[42].

Programmed ICI was the first modality introduced as a penile rehabilitation strategy after RP by Montorsi et al.[43]. In a randomized study, which consisted of ICI of alprostadil three times a week for 12 weeks, 67% (8/15) of patients reported return of spontaneous erections satisfactory for sexual intercourse compared to the group without any erectogenic treatment at 6 months[43]. The limitations of this study are the small amount of patients, lack of blinding with respect to treatments, and a short period of follow-up.

In another nonrandomized, observational study of post-RP patients presenting for treatment with sildenafil refractory ED, patients were either offered ICI rehabilitation (R) (n = 58) or no rehabilitation (NR) (n = 78)[35]. The patients self-selected their therapy. ICI was suggested triweekly. Only patients who presented within 6 months post-RP, who completed the IIEF questionnaire, and who had been followed for at least 18 months were included. At 18 months post-RP, there were statistically significant differences between the two groups in the percentage of patients who were capable of having medication-unassisted intercourse (R = 52% vs. NR = 19%), mean erectile rigidity (R = 53% vs. NR = 26%), mean IIEF-EF domain scores (R = 22 vs. NR = 12), and the percentage of patients responding to ICI (R = 95% vs. NR = 76%). Although supportive of the concept of early penile rehabilitation, this study suffers from a strong patient self-selection bias and lack of a placebo control arm. Unfortunately, due to the perceived invasiveness of ICI therapy, it is difficult to convince patients to self-inject frequently enough to benefit from this rehabilitative therapy.

### Vacuum Erection Device

VED therapy has been around for more than a century and has continued to assume a role in the on-demand management of men with post-RP ED. More recently, it has been investigated as rehabilitation therapy. Raina et al.[44] compared 74 patients (group 1) who used early VED daily for 9 months and 35 patients (group 2) who were observed without any erectogenic treatment. Men and their partners were mailed questionnaires. The results were inconclusive; 19/60 patients (32%) reported return of erections at 9 months, with 10/60 (17%) having erections sufficient for sexual intercourse. In group 2, only 4/35 of these patients (11%) had erections sufficient for successful vaginal intercourse and the rest of the patients (26%) sought adjuvant treatment. The duration of the VED application was not specified, although the constriction band was used only for intercourse. Although VED is effective in the treatment of post-RP ED, it has not yet been proven to be effective in penile rehabilitation protocols.

Köhler et al.[45] analyzed 28 men who were randomized to early VED or a control group. The VED group commenced therapy 1 month after RP, whereas the control group had VED instituted 6 months after RP. Postoperative sexual health inventory for men (SHIM) scores based on spontaneous function were higher in the treatment group (12.4) at 6 months than that in the control group (3.0). Furthermore, in the treatment group, no significant changes in stretched flaccid penile length were measured at 3 or 6 months postoperatively. In the control group, the mean penile length loss at 3 and 6 months was approximately 2 cm.

Determination of possible benefits, efficacy, and utilization of VED therapy in penile rehabilitation programs awaits further studies. Also, further study will be required to optimize the therapeutic regimen.

An additional criticism for VED as a rehabilitation method is its unknown mechanism in improving spontaneous erections.

### NEUROMODULATION

The clinical potential of neuromodulatory therapy is based on the recognition that although the peripheral nervous system demonstrates an intrinsic ability to regenerate after injury, this endogenous response is somewhat limited and does not usually produce a full recovery of function[46]. ED remains a common cause of significant postoperative morbidity for men undergoing radical therapies for prostate cancers or other pelvic malignancies, resulting from the CN being inadvertently axotomized, lacerated, or stretched at the time of surgery[32]. Neuroprotective interventions would be particularly useful in order to address a variety of possible mechanisms contributing to CN injury, including inflammation, oxidative stress, immunologic responses, ischemia, excitotoxicity, lipid peroxidation, free radical production, and apoptosis[47]. There are several options that are under investigation for neuromodulation (Tables 2 and 3).

**TABLE 2**
**Medical Therapies for Improving Erection Recovery**

| | Putative Mechanisms of Action |
|---|---|
| Corticosteroids | Anti-inflammatory action |
| Immunophilin ligands | Immune modulation |
| | Antiapoptosis |
| Erythropoietin | Antiapoptosis |
| | Cryoprotection and/or neurogenesis |
| Statins | Decreased LDL cholesterol in endothelial cells |
| | Down-regulation of RhoA/Rhokinase |
| | Up-regulation of NO synthase |
| | Increased NO bioavailability |

**TABLE 3**
**Surgical Therapies for Improving Erection Recovery**

| | Putative Mechanisms of Action |
|---|---|
| Implantable CN electrode | Enhanced expression of regeneration |
| | Increased brain-derived neurotrophic factor |
| Cavernous nerve grafting | Neurotrophic factor |
| | Axonal reconstruction |

#### Corticosteroids

Two reports have appeared in the literature that examine whether corticosteroids improve ED outcomes by modifying the acute postoperative inflammatory response to CN after RP. A 6-day course of methylprednisolone was used in a placebo-controlled, randomized study of 70 men undergoing NSRP[48]. The medication was started 16–22 h after surgery. A similar study was done with the intraoperative local administration of betamethasone cream 0.1% on the area of the CN in 60 men[49]. Neither study demonstrated appreciable improvement in erection recovery after 12 months follow-up.

### Immunophilin Ligands

Immunophilin ligands represent an exciting new class of agents with well-characterized, preclinical neuroprotective and neuroregenerative properties[46]. The neurotrophic characteristics of immunophilin ligands hold potential for the treatment of many urological and nonurological neurotraumatic or neurodegenerative conditions, including spinal cord injury, peripheral neuropathies, and ED following radical pelvic surgeries[50]. Immunophilin ligands, which include cyclosporine and FK506 (also known as tacrolimus), bind to immunophilin receptors, cellular signaling proteins present in immune and neural tissue. Using models of CN crush injury in the rat, tacrolimus was found to preserve function, reduce neural degeneration, and stimulate axonal regrowth[50,51].

Lagoda et al.[51] studied the mechanism of FK506 and sildenafil on the EF recovery after CN injury in rats. After unilateral CN crush, rats were treated with sildenafil (20 mg/kg) three times a day for 7 days and FK506 (5 mg/kg) once daily for 5 days. At day 14, EF was measured by electrical stimulation of the CN. Sildenafil and FK506 significantly improved EF as measured by the maximum intracavernous pressure (ICP).

Burnett et al.[52] demonstrated the potential clinical efficacy of the immunophilin ligand GPI 1485 in men who underwent bilateral NSRP. In a multicenter, randomized, placebo-controlled study, conducted between September 2003 and February 2005, controlled multiple fixed-dose GPI 1485 (400 and 1000 mg) was administered to men undergoing NSRP with normal preoperative EF. There was a primary analysis in men 40–59 years old (n = 182) and a secondary analysis in men 60–69 years old (n = 45). Data captured were IIEF-EF Domain, Health Related Quality of Life (HRQOL), Questionnaires (Sexual Function-12 [SF-12] and the UCLA Prostate Cancer Index Short Form) at 3 and 6 months postsurgery. There was a profound decrease from baseline in all domains of the IIEF at 3 and 6 months. There was no difference between the treatment groups and placebo in the 40- to 59-year-old group[52]. The treatment arm in the older-age group showed a decrease over placebo in EF domain at 6 months, although the numbers were small and the difference was not significant. Although this trial did not demonstrate a short-term neuroprotective benefit from the neuroimmunophilin ligand, it is possible that the study did not follow the outcome long enough. Ongoing trials (phase IV, randomized, double-blind, placebo-controlled) are in progress to evaluate the safety and efficacy of FK506 in the prevention of ED in men following bilateral NSRP[53].

### Erythropoietin

Erythropoietin (EPO) is a cytokine hormone that stimulates erythropoiesis under hypoxic conditions. Recently, EPO and its receptor have been found to be abundantly expressed in the central and peripheral nervous systems[54]. EPO has been shown to be protective in organs unrelated to the nervous system, such as the heart and kidney. Protective effects independent of neuroprotection, such as protection via the endothelium, are potentially plausible, but they require further investigation[55]. Liu et al.[56] described EPO receptor immunoreactivity in penile tissue and prostate specimens, confirming its localization to the periprostatic ganglia of the neurovascular bundle, the penile dorsal nerves, and sinusoidal endothelium of the corpus cavernosum[56]. This suggested a likely role for endogenous EPO within these tissue and provided the rationale for its clinical evaluation as a protective agent locally[56]. Burnett et al.[57] evaluated retrospectively the potential benefit of EPO administered to preoperatively potent patients undergoing NSRP (40,000 IU subcutaneously, single injection on preoperatively day). PDE5i "on-demand" use was applied. Potency evaluations were monitored by IIEF-5 administered preoperatively and at 3, 6, and 12 months postoperatively. EPO-treated patients demonstrated significantly higher scores than the control group. At 12 months postoperatively, the patients performing sexual activity were 87 and 68% of EPO-treated and control patients, respectively. This finding further suggests the possible utility of innovative strategies for neuromodulatory therapy.

#### Cavernous Nerve Grafting

Consistent with traditional precepts for surgical treatment of injured peripheral nerves, reconstructive procedures have been used to re-establish CN structural continuity[47]. This possibility has been tested in a rat model of CN ablation. Animals were divided into three groups (rats with genitofemoral nerve graft interposition, CN ablation, and sham surgery). After 4 months, electrical stimulation produced erection in 50% of rats with grafts, 10% in nerve-ablated animals, and 100% in sham-treated rats. These results suggested that CN grafting may be useful in restoring potency after CN injury[58].

Walsh[59] initiated a randomized, blinded, pilot investigation in humans, interposing genitofemoral nerve graft in six men at the time of RP, but he discontinued the investigation after failing to see any difference in erection recovery compared with six control patient after 5 years. Chang et al.[60] found that among 30 preoperative patients who underwent bilateral resection of CN and autologous sural nerve grafting, 18 (60%) were confirmed to have recovered spontaneous erectile ability and 13 (40%) were able to engage in sexual intercourse (seven unassisted and six assisted by sildenafil 50 or 100 mg) at a mean follow-up of 23 months.

Several limitations of CN graft reconstructive surgery have been posed: a more extensive surgery may result in added perioperative morbidity, biochemical properties of the nerve involved in reconstruction may differ from autonomic CN conditions, and conditions of locally advanced disease may require adjuvant therapies that could impact on the eventual viability of the CN graft[47,61].

#### Implantable CN Electrode

Burnett et al.[62] explored the feasibility of using an implantable electrode array for CN stimulation for patients undergoing NSRP. The implantable CN electrode array was placed over the neurovascular bundles in 12 patients undergoing open retropubic RP. Six of 12 (50%) patients demonstrated a significant increase in penile circumference after CN stimulation. Lack of response in some subjects may have related to differences in response to anesthesia, as anesthetics agents are known to suppress tumescence. The findings suggested that an implantable system is a feasible therapeutic option to improve post-RP penile erection[62].

### NEW DIRECTIONS

New drugs or interventions would be particularly useful in order to address a variety of possible mechanisms contributing to recovery of EF.

#### Statins

Statins, which have an important part in the secondary prevention of cardiovascular diseases, are known to improve endothelial function by decreasing the action of LDL cholesterol on endothelial cells and up-regulating endothelial NO synthase expression, leading to improved NO bioavailability[63]. Hong et al.[64] reported that in a prospective, randomized, placebo-controlled trial, potent men without hypercholesterolemia, who were undergoing bilateral NSRP for clinically localized prostate cancer and who received atorvastatin at a dose of 10 mg daily from postoperative days 1–90, had a significantly higher postoperative IIEF-5 score than controls at 6 months postoperatively ($p$ = 0.003). These data suggest that postoperative treatment with atorvastatin in men who report normal EF preoperatively may contribute to earlier recovery of EF after NSRP.

### Regenerative Medicine

Concepts of tissue reconstruction have been applied to neural components of the erection apparatus with the supposition that tissue engineering or stem cell therapy may have roles in the face of CN injury[65]. While this application has caused enormous excitement, much more scientific investigation is required to establish therapeutic utility.

### Gene Therapy

The genetic modification of differentiated target cells, as it is applied to the penis for promotion of penile erection, has offered another therapeutic strategy[66]. A phase I clinical trial in which the maxi-K gene was delivered by DNA plasmid has shown safety effects and possible benefits[67]. This clinical trial consisted of only a small number of patients and did not include a control arm. Thus, definitive judgments about the success of gene therapy in humans to treat ED remain limited. However, this study provided enough interest to stimulate further investigation in this area.

### Anti-Inflammatory Agents

Anti-inflammatory agents may be proposed to have a potential role in protecting cavernous tissue. One possibility to consider is annexin A1 (ANXA1), a glucocorticoid-activated member of the annexin family[68]. It is considered an important endogenous anti-inflammatory mediator, which is activated in response to cellular or tissue injury[69,70]. ANXA1 treatment protected against experimental splanchnic, myocardial, renal, and cerebral I/R injury[71,72]. Further investigation may also reveal the efficacy of ANXA1 for promotion of penile protection after CN injury.

## CONCLUSION

In summary, major objectives exist today to preserve sexual function outcomes, while meeting oncological management goals for all prostate cancer treatments. In moving forward at this time, it is important to understand risk factors for ED and pathogenic mechanisms associated with this condition. Current standard on-demand treatments may be offered according to a stepwise treatment algorithm. At the same time, it is also understood that novel treatments currently under study, such as erection rehabilitation and neuromodulatory therapies, may best achieve the ideal outcomes of restored or preserved natural EF. Consideration should be given to developing such perioperative interventions adjunctively in order to improve functional outcomes even further and maximize preservation of quality of life.
